# Fabrication of a Low Adhesive Superhydrophobic Surface on Ti6Al4V Alloys Using TiO_2_/Ni Composite Electrodeposition

**DOI:** 10.3390/mi10020121

**Published:** 2019-02-13

**Authors:** Jianbing Meng, Xiaojuan Dong, Yugang Zhao, Rufeng Xu, Xue Bai, Haian Zhou

**Affiliations:** School of Mechanical Engineering, Shandong University of Technology, Zibo 255000, China; dongxiaojuan@sdut.edu.cn (X.D.); zhaoyugang@sdut.edu.cn (Y.Z.); xurufeng2003@126.com (R.X.); lz8016@126.com (X.B.); zhouhaian@sdut.edu.cn (H.Z.)

**Keywords:** superhydrophobic, low adhesive, Ti6Al4V, composite electrodeposition

## Abstract

A superhydrophobic surface with low adhesion and good wear resistance was fabricated on Ti6Al4V substrates via TiO_2_/Ni composite electrodeposition, and subsequently modified with a fluoroalkylsilane (FAS) film. Scanning electron microscopy (SEM), energy dispersive spectroscopy (EDS), and optical contact angle measurements were used to characterize the surface morphologies, chemical compositions, and surface wettability. The superhydrophobicity of the as-prepared surface results from the fabrication of a hierarchical structure and the assembly of low-surface energy fluorinated components. The as-prepared surface had a water contact angle as high as 162.6° and a sliding angle close to 1.8°. Scratch and abrasion tests showed that the superhydrophobic coating provided a superior wear resistance and stable mechanical abrasion protection. In addition, the influence of processing conditions, such as working voltage, deposited time, pH value, and TiO_2_ concentration, was also investigated.

## 1. Introduction

A titanium alloy is one of the most attractive engineering materials for aerospace, aircraft, and biomedical applications, because of its high specific strength, good corrosion resistance, and excellent heat resistance [1,2]. Recently, superhydrophobic surfaces on titanium alloys with a water contact angle greater than 150° and a sliding angle lower than 10° have aroused much attention. They can provide widespread utilizations in anti-icing, anti-adhesion, and oil/water separation [3,4,5]. Although superhydrophobic surfaces have been proved to be effective in improving the corrosion resistance of titanium alloys, their low hardness, poor wear resistance, and high friction coefficient prevent them from being extensively employed in engineering applications [6,7]. 

Generally, the special wettability of the superhydrophobic surface can be attributed to the coordination of the surface hierarchical structure and low surface free energy material. Therefore, artificial superhydrophobic surfaces are generally prepared via the combination of the creation of a hierarchical rough structure and the reduction of surface free energy using a low surface energy material. There are various methods for the fabrication of superhydrophobic surfaces, e.g., oxidation, anodization, etching, deposition, etc. Guo et al. developed a thermal oxidation and immersion method to fabricate a superhydrophobic film with a good chemical stability [8]. Zhang et al. prepared a stable superhydrophobic film on a titanium substrate via an electrochemical oxidation method. The films have a long-term superhydrophobic durance and excellent anti-corrosive property [9]. Gao et al. presented an anodic oxidation method to obtain superhydrophobic titanium alloy surfaces with a low roughness and good abrasion resistance [10]. Hizal et al. modified an electrochemical anodizing process to create 3D nanopillared TiO_2_ nanostructures on a titanium substrate, and explored bacteria adhesion properties of as-prepared, as well as LbL-coated, substrates. The hierarchical nanostructuring of titanium and the subsequent coating of resulting topographical features with a self-defensive, antibacterial LbL film enabled a synergistic action of hierarchical nanotopography [11]. Lu et al. fabricated superhydrophobic titanium surfaces via environmentally friendly electrochemical etching [12]. Prepared surfaces have water contact angles of more than 150° and rolling angles of less than 2°. Wang et al. constructed superhydrophobic surfaces on Ti6Al4V substrates using an immersion and deposition method [13]. Pb-deposited surfaces display a good corrosive resistance and excellent self-cleaning property with a water contact angle of 165.5° and a sliding angle of 4.6°. 

Chemical etching is an inexpensive and relatively simple method that can be easily scaled-up. However, due to the use of various corrosive acids, such as phosphoric, oxalic, and sulfuric acids, this approach is not an environmentally friendly procedure. In comparison with oxide layers developed from chemical etching, anodic films are less inclined to crack and peel from age due to a greater force and adhesion. Although the anodization method is one of the well-established approaches for generating dual hierarchical structures for superhydrophobic surfaces, the as-prepared sample has to be heated at about 1000 °C. In addition, chemical deposition is an effective approach for fabricating superhydrophobic coatings on titanium alloys. Unfortunately, the deposited superhydrophobic surface shows a poor chemical stability and mechanical durability. Therefore, it is necessary to develop methods for fabricating the superhydrophobic surface with a superior wear resistance and mechanical durability.

Electrodeposition is considered an effective technique for fabricating artificial superhydrophobic surfaces because of its low cost, scalability, simplicity, and reproducible process that permit its use for a range of applications. Moreover, the formation of dual scale hierarchical structures can be easily controlled by varying the electrodeposition parameters, such as deposition voltage, processing time, reactant concentration, and so on. There are reports about enhancing the chemical stability and mechanical durability of superhydrophobic electrodeposits. However, most of them are concentrated on the metals Al, Cu, Zn, and Mg and their alloys [14,15,16]. Unfortunately, to the best of our knowledge, there are few reports about the preparation of superhydrophobic surfaces on titanium alloys using electrodeposition. Although He et al. presented an effective and economic approach for the fabrication of SHP Zn/ZnO coating on a titanium substrate simply by utilizing electrodeposition and anneal treatment, the deposited samples were placed in a watch glass with filter paper and annealed at 200 °C [17]. In addition, the hardness and wear resistance of the obtained superhydrophobic coating was not satisfied. 

In order to improve the hardness and wear resistance of superhydrophobic surfaces, a composite electrodeposition method has been developed in the current literature [18]. Many particles with different properties can be employed in this approach, resulting in the enhancement of abrasion resistance, tribology, and corrosion resistance. In this study, superhydrophobic surfaces on Ti6Al4V substrates were fabricated by TiO_2_/Ni composite electrodeposition. The conditions of prepared surfaces with micro/nano hierarchical structures were measured, and a detailed evolution of surface properties was studied. The surface morphology, chemical compositions, surface wettability, water repellence, and wear resistance of the as-prepared films on Ti6Al4V substrates were characterized by a scanning electron microscope (SEM), energy dispersive spectroscopy (EDS), water contact angle measurements, and a dry wear tester. 

## 2. Materials and Methods 

### 2.1. Materials and Preparation

Commercially available Ti6Al4V (Fe 0.3%, C 0.1%, N 0.05%, H 0.015%, O 0.20%, V 3.5–4.5%, Al 5.5–6.8%, Ti 90.0%) was used as the specimen material for the electrodeposition of functional surfaces. Ti6Al4V plates, with a size of 40 mm × 30 mm × 2 mm, were polished with metallographic abrasive papers (from 100 to 1500 grades). As-abraded specimens were ultrasonically degreased in anhydrous alcohol for 5 min, followed by ultrasonic cleaning with deionized water for 10 min, before being dried completely. In order to avoid contamination and mitigate the effects of the reagent concentration, deionized water was used to clean the specimen before each process.

### 2.2. Experimental Procedure

Electrolytic solution was prepared by adding 250 g NiSO_4_·6H_2_O, 50 g C_6_H_5_O_7_(NH_4_)_3_, 25 g Na_2_SO_4_, 30 g C_2_H_4_O_2_, 0.05 g C_12_H_25_SO_4_Na, and 100 g NH_3_·H_2_O into deionized water under magnetic stirring. Nano-TiO_2_ was added into deionized water to prepare slurry, which was uniformly dispersed in the liquid by mechanical stirring. Then, the slurry was added into the electrolytic solution, followed by ultrasonic vibration. The Ti6Al4V plate and a copper plate of equal size were taken as the cathode and anode, respectively, with a distance of 20 mm. A programmable AC/DC power supply was applied to the two electrodes, with different voltages ranging from 5 V to 30 V.

**Figure 1:**
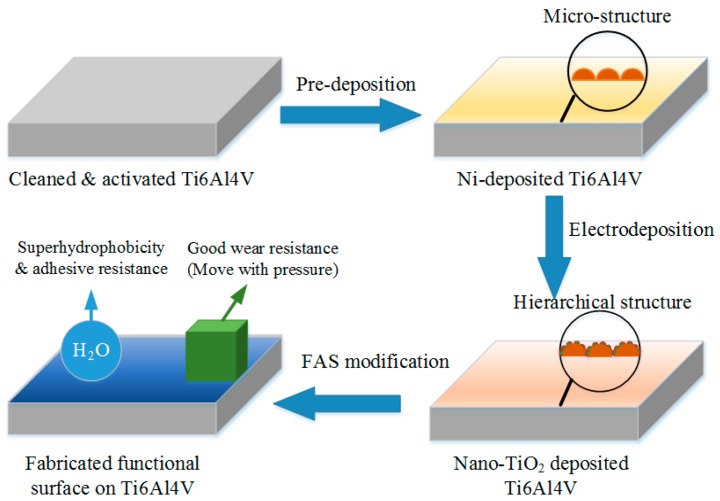
Schematic diagram of the fabrication of functional surfaces on Ti6Al4V substrates.

Preparation of functional surfaces is based on the combination of micro/nano rough structures and low-energy chemical compositions. In this study, it can be divided into the construction of hierarchical structures and the surface modification of FAS, as shown in Figure 1. For activating the Ti6Al4V specimen, it was immersed in cleaning solution and activating solution (No. 1 and No. 2), respectively. The cleaning solution was prepared by adding 20 g NaOH, 25 g Na_2_CO_3_, and 50 g Na_3_PO_4_·12H_2_O into 1 L deionized water. Activation solution No. 1 was composed of 150 g NaCl and 20 g HCl (36%). Activation solution No. 2 was obtained by adding 5 g NiCl_2_·6H_2_O, 90 g C_6_H_8_O_7_·H_2_O, and 150 g C_6_H_5_Na_3_O_7_·2H_2_O into deionized water. In addition, a pre-deposition was indispensable for a better bonding strength between the coating and the substrate. The pre-deposition solution was prepared by adding 400 g NiSO_4_·6H_2_O, 10 g NiCl_2_·6H_2_O, 70 g CH_3_CH_2_COOH, and 20 g HCl (36%) into deionized water. After electrodeposition, the specimens were immersed in a 1.0 wt % FAS (tridecafluoroctyltriethoxysilane, C_8_F_13_H_4_Si(OC_2_H_5_)_3_) in ethanol for 60 min and then dried at 70 °C for 30 min. Finally, the superhydrophobic and low adhesive surfaces with hierarchical micro/nano structures were obtained on the Ti6Al4V alloy substrates.

### 2.3. Characterization

The morphology of as-prepared sample surfaces was observed by scanning electron microscopy (SEM, FEI Sirion 200, Hillsboro, OR, USA). The corresponding chemical compositions were characterized by energy-dispersive spectra (EDS, INCA Energy, Oxford, UK). The water contact angles were measured with an optical contact angle measuring instrument (DSA 100, Kruss, Hamburg, Germany). The sliding angles were measured using the conventional sessile-drop method A 5 μL deionized water droplet was dropped on the obtained surface, and the average of three measurements of different positions was regarded as the final contact angle. The angle at which the water droplet initiated rolling off the tilted surface was defined as the water sliding angle. The micro-hardness was measured with a nano-hardness tester (NanoTest Vantage, Micro Materials, Wrexham, UK). In addition, wear testing was performed using a dry wear tester (MMS-2A, Jinan HengXu, Jinan, China) at room temperature. 

## 3. Results and Discussions

### 3.1. Surface Morphology

**Figure 2:**
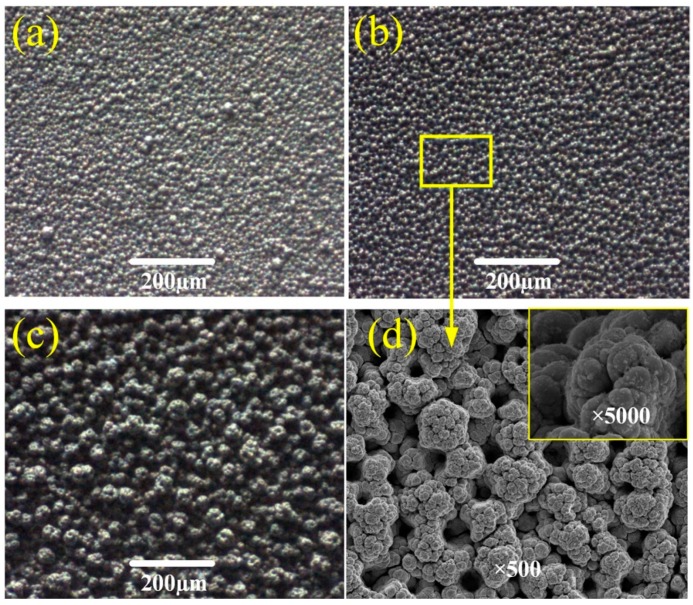
Images of surface morphology of samples electrodeposited in the TiO_2_ concentration of 7 g/L for 5 min at the pH value of 4 and different working voltages. (**a**) 20 V; (**b**) 25 V; (**c**) 30 V; (**d**) 25 V with ×500 and ×5000 magnifications.

Figure 2 shows the SEM images of Ti6Al4V sample surfaces electrodeposited in a 7 g/L TiO_2_ concentration for 5 min at a 4 pH value and different working voltages. It can be seen that the working voltage significantly influences the surface morphology. The higher the working voltage is, the greater the current density becomes, along with the rise of the electrodeposition of TiO_2_ particles. When the working voltage increases to 20 V and 25 V, more and more nano-scale bumps appear on the micro-scale columns and form the micro/nano hierarchical structure. In Figure 2a, the hierarchical structure unevenly distributes on the as-prepared surface. When the working voltage increases to 25 V, the hierarchical structure becomes obvious and well-distributed, as shown in Figure 2b. In addition, with the working voltage increased to 30 V, the sizes of the rough hierarchical structures greatly increase, and even link together into block-like structures, as shown in Figure 2c. Figure 2d shows the two-level structures with ×500 and ×5000 magnifications of the as-prepared surfaces at the working voltage of 25 V. In Figure 2d (×500 magnification), it can be seen that the TiO_2_/Ni deposited surface is covered with rough structures with a size of about 15 μm, resulting in the micro column-like structures. When further magnified by ten times, some nano bump-like structures, with a size ranging from 20 nm to 50 nm, are stacked and lump together on the column-like structures. Compared with the traditional flower-like structures, the micro columns are analogous to the papillae, generating the first level of the hierarchical structure. Then, nanoparticles are similar to the bead-like crystals, providing the second level of the hierarchical structure.

**Figure 3:**
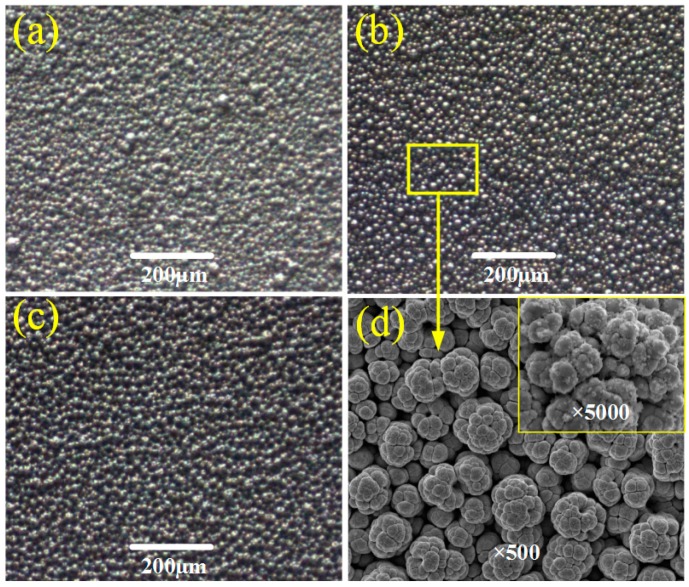
Images of surface morphology of samples electrodeposited in the TiO_2_ concentration of 7 g/L at the working voltage of 25 V and the pH value of 4 for different processing times. (**a**) 4 min; (**b**) 5 min; (**c**) 6 min; (**d**) 5 min with ×500 and ×5000 magnifications.

Figure 3 shows the SEM images of the sample surfaces electrodeposited in the TiO_2_ concentration of 7 g/L for different times at the working voltage of 25 V and the pH value of 4. It can be seen that the processing time is also a key factor affecting the surface morphology. When the deposition time is 4 min, more micro columns appear, but are unevenly distributed on the sample surface, as shown in Figure 3a. It can be explained that TiO_2_ particles are deposited preferentially on the grains’ edges of the Ni coating due to the “tip effect”. The micro structures on the columns provide a crucial nucleus for the deposition of nano TiO_2_ particles. Moreover, as time goes by, TiO_2_ particles tend to grow up and reunite to form the hierarchical structures on the as-prepared surface, as shown in Figure 3b. However, once the processing time exceeds 5 min, columns begin to aggregate into larger block-like structures, resulting in degradation of the hierarchical structure, as shown in Figure 3c. Figure 3d shows the surface morphology with ×500 and ×5000 magnifications of the as-prepared sample at the processing time of 5 min. It is observed that the as-prepared surface exhibits a typical hierarchical structure. The hierarchical structure is obtained by adding TiO_2_ particles, forming the nano bump-like structure on the micro column-like structure.

**Figure 4:**
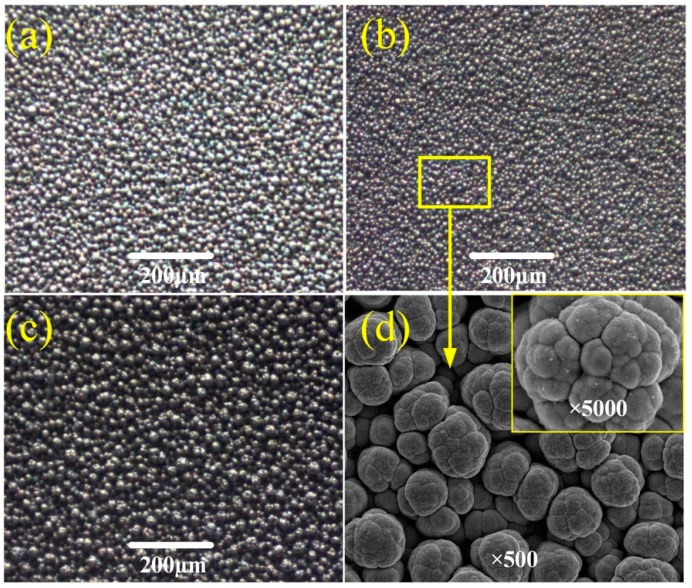
Images of surface morphology of samples electrodeposited in the TiO_2_ concentration of 7 g/L for 5 min at the working voltage of 25 V and different pH values. (**a**) 3; (**b**) 4; (**c**) 5; (**d**) 4 with ×500 and ×5000 magnifications.

Figure 4 shows the SEM images of Ti6Al4V samples electrodeposited in the TiO_2_ concentration of 7 g/L for 5 min at the working voltage of 25 V and different pH values. Generally, lower pH values of the electrodeposition solution may cause serious hydrogen evolution and a low current efficiency, which result in fewer columns being generated on the sample surface per unit time. In this study, the micro column is one of the important factors in the fabrication of the hierarchical structure. In order to obtain the desired hierarchical structure, the columns have to be numerous enough and well-distributed. In Figure 4a, a large number of columns appear on the sample surface with the increase of the pH value. When the pH value is 4, the columns become numerous enough and uniform, as shown in Figure 4b. In addition, with the pH value increased to 5, almost all of the columns overlap and transform into large massive textures, as shown in Figure 4c. It may be explained by the higher pH value causing the oxidation of titanium alloys and the formation of Ti(OH)_2_. Then, these precipitates cover the hierarchical structures and change their appearance into micro block-like structures. Figure 4d shows the surface morphology with ×500 and ×5000 magnifications of the as-prepared sample at the pH value of 4. It can be found that the desired surface morphology attributes to the well-distributed micro column and the uniform nano bump. 

**Figure 5:**
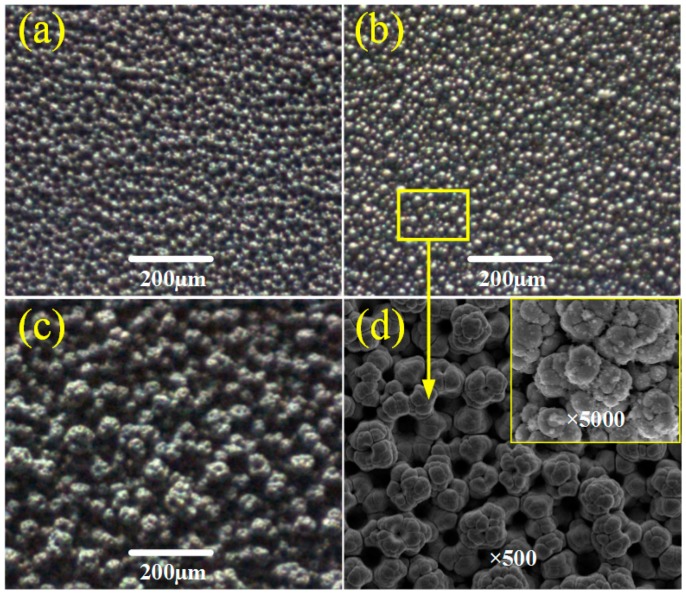
Images of surface morphology of samples electrodeposited for 5 min at the working voltage of 25 V and the pH value of 4 in different TiO_2_ nano-particles concentrations. (**a**) 5 g/L; (b) 7 g/L; (**c**) 9 g/L; (**d**) 7 g/L with ×500 and ×5000 magnifications.

Figure 5 shows the SEM images of the sample surfaces electrodeposited for 5 min at the working voltage of 25 V and the pH value of 4 in different TiO_2_ particle concentrations. It is clear that the as-prepared samples show different surface morphologies. When the TiO_2_ concentration is 5 g/L, the granular TiO_2_ particles grow up and change their appearance to one which is bump-like, as shown in Figure 5a. With the increase of the TiO_2_ concentration, these bumps become uniform and fabricate the desired hierarchical structures with the micro columns, as shown in Figure 5b,d. However, these nano bumps tend to grow up and reunite to form micro-scale coralloid structures with the TiO_2_ concentration increased to 7 g/L, as shown in Figure 5c. The emergence of coralloid structures leads to the disappearance of the nano bumps and the degeneration of the required hierarchical structures.

### 3.2. Chemical Composition

**Figure 6:**
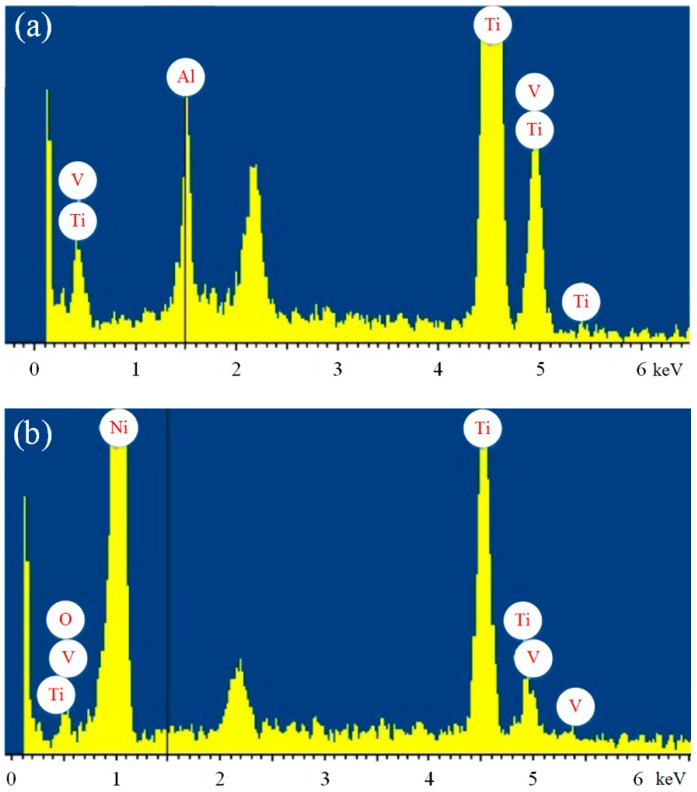
EDS spectra of Ti6Al4V alloy surfaces before (**a**) and after (**b**) TiO_2_/Ni composited electrodeposition without FAS modification.

Chemical compositions of Ti6Al4V surfaces can be measured with EDS spectra. Figure 6a,b are the EDS regional analysis results of untreated specimen surfaces and TiO_2_/Ni deposited coatings, respectively. Comparing the untreated specimen surface with the TiO_2_/Ni deposited surface before FAS modification, the main elements, such as Ti and V, are detected on both surfaces. However, it is clear that no Al peaks are found on the deposited surface. It can be explained that during the process of composite electrodeposition, most of the aluminum in the outer layer reacts with hydrochloric acid. Because of the presence of the Ni element, the Ti element has decreased from 90.48 wt % to 62.71 wt %. In addition, the presence of Ni and O in Figure 6b indicates that Ni ions and TiO_2_ nano particles are successfully deposited on the sample. Furthermore, Ni and O elements increase to 28.11 wt % and 5.6 wt %, respectively.

### 3.3. Surface Wettability

**Figure 7:**
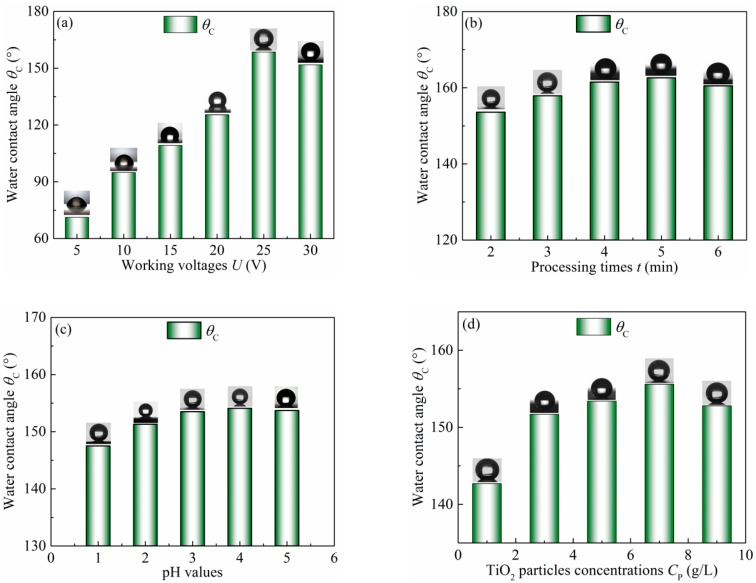
Contact angles of sample surfaces electrodeposited at different process parameters, such as (**a**) working voltage, (**b**) processing time, (**c**) pH value, and (**d**) TiO_2_ concentration.

A series of experiments were carried out to investigate the influence of processing parameters, such as working voltage, deposited time, pH value, and particles concentration, on the wettability of TiO_2_/Ni deposited surfaces comprehensively, as shown in Figure 7.

Figure 7a shows the wettability of the sample surfaces electrodeposited in the TiO_2_ concentration of 7 g/L for 5 min at the pH value of 4 and different working voltages ranging from 5 to 30 V. The water contact angle significantly rises with the increase of the working voltage, reaches the highest value of 158.6° at 25 V, and then decreases slightly but remains above 150° at 30 V. Consequently, the above result shows that the wettability of the as-prepared surface switches from hydrophilic to hydrophobic or even superhydrophobic. At a lower working voltage, there is no uniform current density distribution on the sample surface. A non-uniform current density hampers the growth of the crystal nucleus. With the increase of working voltages, the distribution of current density tends to be uniform, which leads to the increase of the crystal nucleus. Eventually, some micro columns and nano bumps grow significantly. The highest water contact angle obtained at 25 V may attribute to the dual scale structure. As the working voltage increases up to 30 V, the hierarchical structures tend to be connected in only micro block-like structures, resulting in a decrease of the water contact angle. 

The processing time also has a great importance in regards to the wettability of the as-prepared sample surface. The Ti6Al4V sample was electrodeposited in the TiO_2_ concentration of 7 g/L at the working voltage of 25 V and the pH value of 4. The results are shown in Figure 7b. The water contact angle rises from 153.6° to 162.6° along with prolonging the time from 2 to 5 min, and exhibits a slight decrease to 160° with the processing time increased to 6 min. The longer the processing time is, the rougher the surface becomes along with the rise of the water contact angle. However, as the processing time increases up to 6 min, big block-like structures occur from the aggregation of some columns and bumps, resulting in a slight decrease of the water contact angle. 

Interestingly, the increase of the pH value results in the transformation of surface wettability. Figure 7c shows the effect of pH value on the water contact angle when the working voltage is 25 V, the processing time is 5 min, and the TiO_2_ concentration is 7 g/L. The water contact angle is 147.5° when the pH value is 1. As the pH value increases to 3, the water contact angle gradually increases to 153.5°. Below the range of 3–4, it reaches the highest value of 154.1°. However, the water contact angle decreases slightly to 153.7°. It is well-known that the hierarchical structure plays an important role in determining the surface wettability. In fact, the lower pH value leads to coarse grain, lower porosity, and poor hierarchical structures. In contrast, under the higher pH value, the Ti6Al4V sample is oxidized to form precipitates, which fill in the gaps between the bumps and reduce the hierarchical structures, resulting in the decrease of the water contact angle.

In addition, the effect of TiO_2_ concentration on surface wettability was also investigated at the working voltage of 25 V and the pH value of 4 for 5 min. The results are shown in Figure 7d. Varying the TiO_2_ concentration from 1 to 5 g/L, the water contact angle markedly increases from 142.7° to 153.4°. It reaches the maximum value of 155.6° when the TiO_2_ concentration is 7 g/L. As the TiO_2_ concentration increases to 9 g/L, the water contact angle decreases evidently to 152.8°. Actually, when the TiO_2_ concentration is relatively low (1 g/L), there are enough nano bumps to form the micro/nano hierarchical structure. However, when the TiO_2_ concentration is relatively high (9 g/L), many bumps assemble together and form microscale corralloid structures. Both above surface morphologies cannot provide enough roughness to make surfaces superhydrophobic in comparison with the hierarchical structure.

As shown in Figure 7, the water droplet on the coated surfaces obtained by the electrodeposition and FAS-modification shows nearly a spherical shape. According to this wetting behavior, the deposited surface with hierarchical structures can catch air and decrease the contact area of the solid-liquid interface. This result can be explained by the Cassie-Baxter theory [19]. In this model, air fills and occupies the hollow space of the hierarchical structures, resulting in the water droplets being suspended on the surface. Thus, the combination of a dual hierarchical structure and a layer with low surface energy can contribute to the low wettability.

### 3.4. Surface-Water Adhesion

Low adhesive superhydrophobic surfaces have extensive applications in engines, tire molds, electrosurgical instruments, and so on [20]. Generally, there exist two factors, including sliding angles and surface energy, determining surface adhesion forces [21,22]. Low surface energy can significantly decrease the surface adhesion force. However, all Ti6Al4V samples are modified by FAS in this study, so the surface energy is the same among all samples. Adhesion force is only considered by sliding angles. 

**Figure 8:**
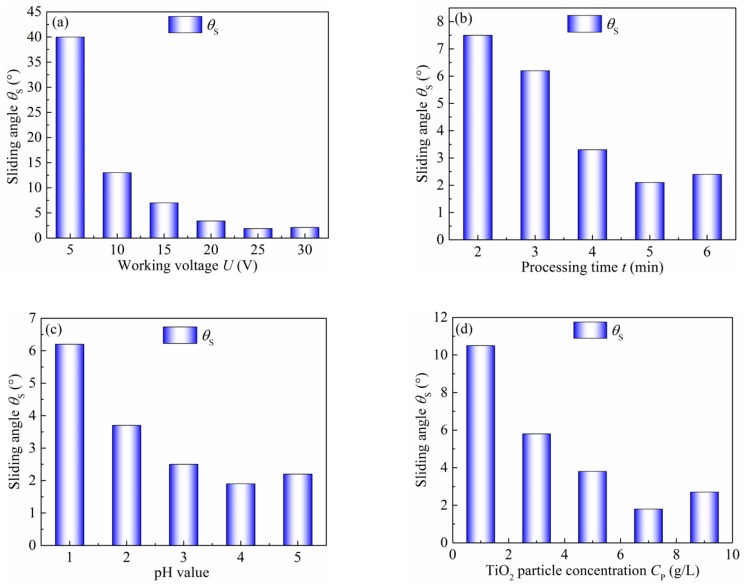
Sliding angles of sample surfaces electrodeposited at different processing parameters, such as (**a**) working voltage, (**b**) processing time, (**c**) pH value, and (**d**) TiO_2_ concentration.

The effects of the working voltage, processing time, pH value, and TiO_2_ particles concentration on the sliding angle were investigated in detail. Figure 8a shows the relationship of the working voltage with the sliding angles. The sample surface was electrodeposited for 5 min in the 7 g/L TiO_2_ particles concentration at a 4 pH value. The working voltage ranged from 5 to 30 V. As shown in Figure 8a, the sliding angle decreases with the increase of the working voltage. This can be explained by the changes of surface morphology. Increasing the working voltage, micro columns and nano bump-like structures increase per unit time, and the contact area of the liquid-solid interface decreases. Consequently, the higher the working voltage, the smaller the sliding angle, and the lower the surface adhesive force. 

In addition, the processing time has a great influence on the adhesion force of the as-prepared sample surface. Figure 8b shows the effect of the deposited time on the sliding angle when the working voltage was 25 V, the pH value was 4, and the TiO_2_ particle concentration was 7 g/L. It can be found that the sliding angle markedly decreases with the extended deposition time, reaches the minimum value of 2.1° after 5 min, and then increases slightly due to the deposition of more TiO_2_ particles on the sample and the formation of hierarchical structures. When the processing time exceeds 5 min, the sliding angle slowly increases.

The pH value is another important processing parameter in the electrodeposition. Figure 8c shows the relationship of the sliding angle with different pH values. The sample surface was electrodeposited for 5 min in the 7 g/L TiO_2_ particle concentration at 25 V. In Figure 8c, the sliding angle markedly decreases from 6.2° to 1.9° with the increase of the pH value from 1 to 4, and then increases slightly to 2.2° when the pH value is 5. It is well-known that the rough hierarchical structure has an important role in determining wettability and adhesion. The lowest sliding angle at a pH value of 4 may attribute to the dual scale structure, which establishes a stable composite solid-liquid-air interface when the as-prepared sample makes contact with water droplets and presents as a Cass-Baxter state. As the pH value increases to 5, the deposited TiO_2_ seems to fill in the gaps between the bumps and reduce the roughness, resulting in a slight decrease of the sliding angle. 

The influence of the TiO_2_ particle concentration on the sliding angle of the sample surface was also investigated at a 25 V working voltage and pH value of 4 for 5 min of depositing time. From Figure 8d, it can be found that the sliding angle gradually decreases with the extension of the concentration of TiO_2_. The as-prepared sample surface has the lowest sliding angle of 1.8°, allowing the water droplet to roll off with ease. This can be attributed to the changes of surface chemical compositions and morphology. Increasing the TiO_2_ concentration can enhance the growth of nano-scale bump-like structures. Hence, the hierarchical structure formed by TiO_2_/Ni composite electrodeposition becomes well-distributed, which leads to the smaller contact area and the lower adhesive force. With the increase of TiO_2_ concentration, the nano-particles grow up and change their appearance from bead-like to block-like. When the TiO_2_ concentration exceeds 7 g/L, more and more block-like structures appear gradually to cover the sample surface. Furthermore, the nano-scale structure disappears and the liquid seems to impregnate between the micro-scale blocks. As a result, the sliding angle begins to increase and it is not easy for the water droplet to roll off.

### 3.5. Surface Wear and Abrasion Resistance

**Figure 9:**
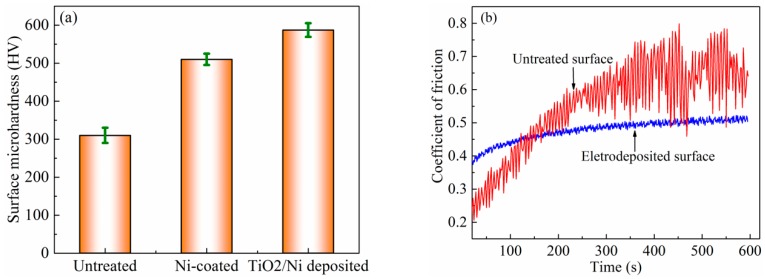
The micro hardness (**a**) and friction coefficient (**b**) of the Ti6Al4V surface before and after TiO_2_/Ni electrodeposition.

In general, the higher the hardness of the material, the better the wear resistance, so the micro hardness is often used as one of the important indicators of surface wear resistance [23]. The surface microhardness was measured using a digital Vickers microhardness tester (HV-1000, China). The values are the average of five measurements at different positions of the Ti6Al4V specimen surface, with a peak load of 0.5 N for 10 s. Figure 9a shows the surface microhardness of the untreated, Ni-coated, and TiO_2_/Ni electro-deposited Ti6Al4V samples. The results demonstrate that the microhardness of the surface increases significantly after electrodeposition. The microhardness of the untreated substrate is only 310 HV, while it increased to 510 HV owing to the formation of a porous Ni-coating on the specimen surface. Subsequently, the surface microhardness increases to 587 HV on the TiO_2_/Ni composite coating. It can be seen that adding nano TiO_2_ particles reduces the free energy of nucleus growth, increases the nucleus density, refines the grain, and makes the hierarchical structures of the composite coating more uniform and fine.

The scratch test is also a feasible way to investigate the abrasion resistance of the functional deposited surface [24,25]. In this study, the friction and wear tests were conducted using a MMS-2A friction and wear tester with block-on-ring friction pairs [26]. The top sample was an immovable as-prepared Ti6Al4V measuring 5 mm × 6 mm × 20 mm. The bottom sample was a rotary WC-Co cemented carbide K30 ring with a Φ40mm outside diameter, Φ16mm inside diameter, and 10 mm thickness. It was performed with a load of 10 N and a constant working speed of 200 rpm for 10 min at room temperature. Figure 9b shows the friction coefficient variation of Ti6Al4V surfaces before and after TiO_2_/Ni composite electrodeposition. The initial friction coefficient of the untreated specimen is only about 0.2, due to some soft phase Al acting as the lubricant in friction press. With the increase of friction time, the surface temperature increases and the soft phase Al is oxidized, which results in the disappearance of lubricants and the increase of the friction coefficient. When the test time exceeds 200 s, the friction coefficient fluctuates greatly. In contrast, the friction coefficient of the electro-deposited surface displays a slight fluctuation and tends to be stable. Its average value decreases from 0.6 to 0.45. This is because the electrodeposited surface has a great microhardness and strong deformation resistance. In addition, the scratch between the as-prepared sample and the rotary ring causes some nano bump-like TiO_2_ to fall off, which can act as a lubricant in the friction process. Compared with the untreated sample, the friction coefficient of the as-prepared sample is stable and the fluctuation range is very small.

**Figure 10:**
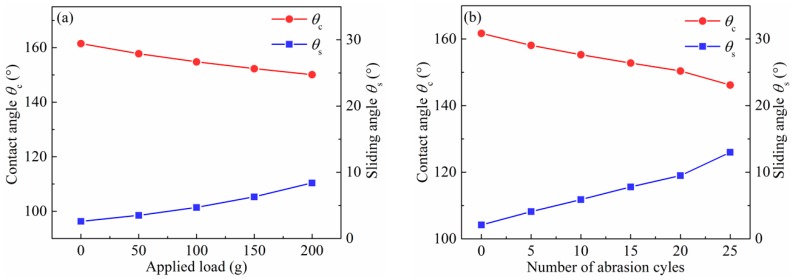
Contact angles and sliding angles of the electrodeposited surfaces after different (**a**) applied loads and (**b**) abrasion cycles of scratch tests.

To further evaluate the mechanical abrasion resistance of the superhydrophobic surfaces, the corresponding tests were carried out using a 1000 grit sandpaper as an abrasion surface, and several different weights as the load for different abrasion cycles. It was tested back and forth, with a distance of 20 cm for each abrasion cycle. Figure 10 shows the changes of contact angles and sliding angles as functions of abrasion loads and cycles. As shown in Figure 10a, the contact angle decreases and the sliding angle increases with the increase of abrasion loads. After 200 g abrasion loads, the surface still maintains its superhydrophobicity, and water repellence with a contact angle of 150.1° and a sliding angle of 8.4°. Figure 10b shows that the contact angle decreases to 150.4° and the sliding angle increases to 9.5° with the increase of the abrasion cycle in 20 abrasion cycles. However, after 25 abrasion cycles, the contact angle decreases to 146.2° and the sliding angle increases to 13°, which cause liquid droplets to hardly roll off the surface. 

**Figure 11:**
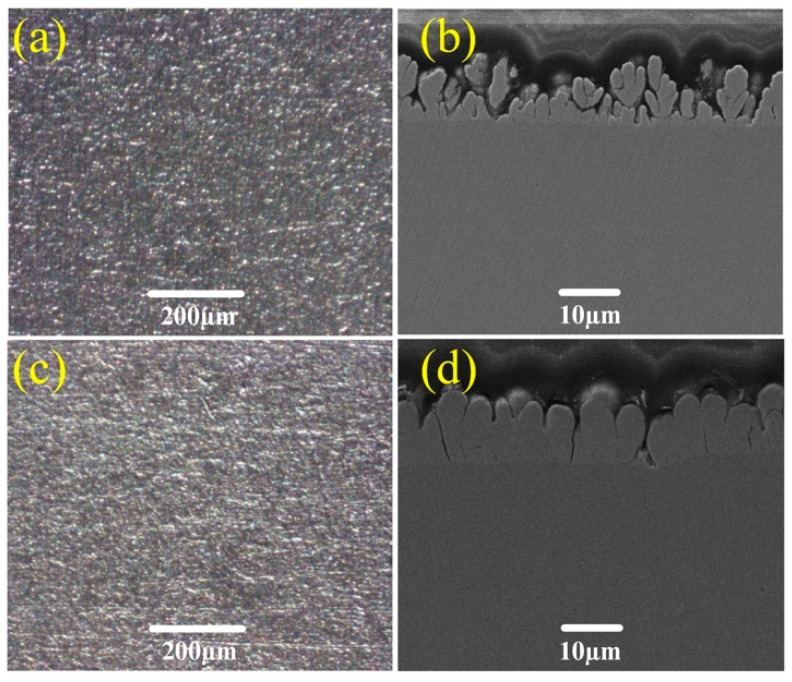
SEM images of surface and cross-section micrograph of the as-prepared sample after abrasion for 20 circles at applied loads of (**a**,**b**) 100 g and (**c**,**d**) 200 g.

Figure 11 shows the SEM images of the as-prepared surfaces after abrasion for 20 circles at an applied load of 100 and 200 g. As shown in Figure 11a, a few shallow scratches are observed on the sample surface, which suggests that the sample surface is slightly scratched by the sandpaper. As the applied load of 200 g is used, the as-prepared surface is featured with severe abrasion wear, as shown in Figure 11c. It seems that some bumps and columns have been destroyed. Due to the decline of the rough hierarchical structures, the water contact angle decreases and the sliding angle increases. Though abrasion makes the bumps on the topper columns wear off, the bumps on the bottom layer still exist, which consist of nano TiO_2_ particles, as shown in Figure 11b. Thus, enhanced wear and abrasion resistance may result from constructing hierarchical structures with micro Ni columns and nano TiO_2_ particles. However, a large applied load causes the as-prepared surface to be worn off, resulting in the loss of the hierarchical structures, as shown in Figure 11d. As a result, the above tests display that the as-prepared surface has excellent wear and abrasion resistance.

## 4. Conclusions

An efficient method was designed to fabricate hierarchical structures on Ti6Al4V alloy surfaces via the TiO_2_/Ni composite electrodeposition method, followed by the modification of FAS. The as-prepared Ti6Al4V surface has a better superhydrophobicity with a water contact angle of 162.6°. Moreover, the electrodeposited coating on the Ti6Al4V substrate has an ultra-low adhesive property with a sliding angle of 1.8°. The micro hardness of the as-prepared surface increases from 310 to 587 HV, and the friction coefficient decreases from 0.6 to 0.45. These results demonstrate that the electrodeposited coating enjoys excellent wear resistance. Abrasion tests with sandpapers further suggest that the as-prepared surface has an outstanding mechanical stability and superhydrophobic durability. The proposed method is feasible and low cost. It can be widely applied for micro/nano devices or systems of superhydrophobic surfaces with excellent water repellence and wear resistance.
